# 39347 Examining the Potential for Tech-Based HIV interventions for Young Black Women

**DOI:** 10.1017/cts.2021.536

**Published:** 2021-03-30

**Authors:** Jaih B. Craddock

**Affiliations:** University of Maryland, Baltimore

## Abstract

ABSTRACT IMPACT: Findings from this study have the potential to improve interventions geared toward YBW, by highlighting the potential for technology-based HIV interventions. OBJECTIVES/GOALS: A stark disparity in HIV exists for Black American women, with 61% of all new HIV infections among American women occurring in Black women. Using technology to address community-level HIV risk may be beneficial, however few studies have examined the association of tech-based communication and HIV prevention behaviors among Black women. METHODS/STUDY POPULATION: Egocentric social network data from 201 young Black women (YBW) were collected from June 2018 to December 2018 to identify how social media use (e.g., amount of time, type of social media used, health information seeking) and sexual health communication (e.g., talk about condom use via face-to-face, text or phone and talk about HIV testing via face-to-face, text or phone) were associated with condom use, HIV testing and interest in pre-exposure prophylactic (PrEP). Statistical analysis proceeded in two stages, descriptive statistics and multivariate logistic regression modeling. RESULTS/ANTICIPATED RESULTS: Instagram (82%) and Snapchat (79%) were the most used social media platforms for communication with SNMs. About 20% of YBW reported spent 4 or more hours on social media per day, and a majority of YBW spoke to at least one SNM via text (85%), face-to-face (98%), or on the phone (97%). Multivariate logistic regression results indicated that YBW who spoke to their SNMs via Instagram had 3.23 times the odds of using condom during last sex, however if they spoke to SNMs on twitter or spent more then 4 hours on social media they had a decrease in odds of using condoms. YBW had 96% decreased odds of ever being tested for HIV if they spoke to a SNM face-to-face about condom use; and notably, YBW had 3 times the increased odds in interest of using PrEP if they had sex with someone they met online or if they sought sexual health information online. DISCUSSION/SIGNIFICANCE OF FINDINGS: By assessing the modes of communication YBW are using to speak with their SNMs and their associations with HIV prevention behaviors, we can better determine the most optimal, efficient, and effective ways of utilizing technology for HIV intervention.

